# Proof-of-Principle Study Suggesting Potential Anti-Inflammatory Activity of Butyrate and Propionate in Periodontal Cells

**DOI:** 10.3390/ijms231911006

**Published:** 2022-09-20

**Authors:** Ana Flávia Piquera Santos, Lara Cristina Cunha Cervantes, Layla Panahipour, Francisley Ávila Souza, Reinhard Gruber

**Affiliations:** 1 Department of Oral Biology, University Clinic of Dentistry, Medical University of Vienna, Sensengasse 2a, 1090 Vienna, Austria; 2 Department of Diagnosis and Surgery, School of Dentistry, São Paulo State University, Aracatuba 16015-050, Brazil; 3 Department of Periodontology, School of Dental Medicine, University of Bern, 3012 Bern, Switzerland; 4 Austrian Cluster for Tissue Regeneration, Donaueschingenstraße 13, 1200 Vienna, Austria

**Keywords:** butyric acid, microbiota, cell culture techniques, periodontium, inflammation mediators

## Abstract

Short-chain fatty acids (SCFAs) are potent immune modulators present in the gingival crevicular fluid. It is therefore likely that SCFAs exert a role in periodontal health and disease. To better understand how SCFAs can module inflammation, we screened acetic acid, propionic acid, and butyric acid for their potential ability to lower the inflammatory response of macrophages, gingival fibroblasts, and oral epithelial cells in vitro. To this end, RAW 264.7 and primary macrophages were exposed to LPSs from *Porphyromonas gingivalis (P. gingivalis)* with and without the SCFAs. Moreover, gingival fibroblasts and HSC2 oral epithelial cells were exposed to IL1β and TNFα with and without the SCFAs. We report here that butyrate was effective in reducing the lipopolysaccharide (LPS)-induced expression of IL6 and chemokine (C-X-C motif) ligand 2 (CXCL2) in the RAW 264.7 and primary macrophages. Butyrate also reduced the IL1β and TNFα-induced expression of IL8, chemokine (C-X-C motif) ligand 1 (CXCL1), and CXCL2 in gingival fibroblasts. Likewise, butyrate lowered the induced expression of CXCL1 and CXCL2, but not IL8, in HSC2 cells. Butyrate further caused a reduction of p65 nuclear translocation in RAW 264.7 macrophages, gingival fibroblasts, and HSC2 cells. Propionate and acetate partially lowered the inflammatory response in vitro but did not reach the level of significance. These findings suggest that not only macrophages, but also gingival fibroblasts and oral epithelial cells are susceptive to the anti-inflammatory activity of butyrate.

## 1. Introduction

Periodontal health is a great importance for the tooth-supporting tissues maintenance composed of the gingiva covering the alveolar bone, where the periodontal ligament fibers are inserted into the cementum layer [1]. The periodontium, however, has to combat permanent attacks coming from the oral cavity, with its bacteria and other sources that may cause harm to the tissue [2]. In particular, it is the virulence factors released by oral pathogens that reach, and may even penetrate, the protective sealing of the junctional epithelium, becoming a major driver of the innate immune response [3]. Fibroblasts and epithelial cells, in turn, release inflammatory mediators to further accelerate the local inflammation [3]. This inflammatory environment is characterized by its proteolytic degeneration of the soft tissue and the osteoclastogenic resorption of the hard tissue [4]. Intrinsic mechanisms to dampen the exacerbating catabolic inflammatory response to virulence factors are necessary to protect the periodontium.

Short-chain fatty acids (SCFAs), including acetic acid, propionic acid, and butyric acid, are released by gut bacteria [5], reaching plasma concentrations in ranges of 25–250 µM, 1.4–13.4 µM, and 0.5–14.2 µM, respectively [6]. SCFAs, apart from serving as an energy source for hepatocytes [7], are also produced by anaerobic bacteria of the periodontal pocket. Oral microbiota producing butyrate and propionate are considered to possess toxic activities in periodontal tissue [8], originally proposed by analyzing dental plaque extracts with mesenchymal cells [9], activation of neutrophil or blocking of macrophage phagocytosis [10,11,12], and inhibition of T-cell proliferation [13]. Later on, the in vivo concentrations of these toxins in the periodontium were identified, making it possible to show a relationship between gingival inflammation and SCFA levels [14]. Severely diseased subjects exhibited propionic and butyric acid levels of approximately 10 and 3 mM, respectively, while these levels did not reach detection limits in healthy sites of mildly diseased subjects [15], and decreasing levels were observed after non-surgical periodontal treatment [16].

There is also reason to assume that SCFAs are a potential intrinsic mechanism to combat the aggravating inflammatory response. For instance, oral administration of *Clostridium butyricum* M588, an enterobacterium producing butyrate, reduced dextran sodium sulfate-induced colitis in rats [17]. Oral administration of berberine in rats increased butyrate level, being associated with less collagen-induced arthritis [18] and periodontal bone loss [19]. Apart from the gut microbiome affecting periodontitis [20], oral microbiota and its metabolites can modulate immunity and alveolar bone homeostasis [21,22]. There is thus uncertainty over whether butyrate and other SCFAs only impede periodontal tissue homeostasis or even support it [23].

Macrophages are involved in periodontal homeostasis. There is solid evidence that butyrate reduces cytokine and nitric oxide production in LPS-stimulated [24,25,26] and in IFNγ-stimulated RAW 264.7 cells [27]. Elsewhere, human monocytes responded with a biphasic increase of IL1, peaking at 2 mM butyrate [13]. We extended this approach towards implementing primary macrophages by pushing the inflammatory response with agonists characteristic for oral inflammation. For instance, fimbriae from *P. gingivalis* increased inflammatory cytokine expression in macrophages [28,29,30]. Furthermore, saliva is a potent inducer of a Toll-like receptor (TLR)-mediated inflammatory response in murine bone marrow macrophages and RAW 264.7 cells [31]. Thus, one aim of this research was to test the impact of SCFAs on the modulation of the inflammatory response of murine bone marrow macrophages to fimbriae and saliva. Considering that previous research was on cytokines, we included the chemokine CXCL2, also called macrophage inflammatory protein 2-alpha, in our bioassay. 

Gingival fibroblasts also respond to SCFAs. Butyrate and propionate inhibit the proliferation of both mouse L929 cells and human gingival fibroblasts [9,32,33] involving apoptosis [34]. Butyrate can increase reactive oxygen species production [33], matrix metalloproteinase activity [35], and TNF-α expression in gingival fibroblasts [36]. Nevertheless, in vitro toxicity and other catabolic effects needs to be interpreted carefully, as, for instance, propionate in drinking water and upon local application onto the joint reduced experimental arthritis and lowered the inflammatory response of synovial fibroblasts [37]. Similar to synovial fibroblasts, gingival fibroblasts are critically involved in periodontitis. Gingival fibroblasts are a potent source of cytokines and chemokines once they are stimulated with IL1β and TNFα [38]. Accordingly, there is a rationale to investigate if butyrate and other SCFA possess anti-inflammatory activity in IL1β- and TNFα-exposed gingival fibroblasts. 

Oral epithelial cells are sensitive to SCFAs [39]. For instance, butyrate decreased intercellular adhesion molecule 1 (ICAM1) expression in the oral squamous carcinoma cell line HSC2 [40] and promoted the migration and invasion of carcinoma cells [41]. Butyrate inhibits the proliferation of HSC3 and HSC4 [42] and the human oral squamous carcinoma cell lines SCC-1 and SCC-9 [43]. Butyrate pushes pyroptosis of gingival epithelial cells [44] and inhibits expression of lymphangiogenic factors in HSC3 cells [45]. Further, in the gingival epithelial Ca9-22 cell line, butyrate- or propionate-exposure cell death accompanied apoptotic and autophagy signaling [46] and the release of damage-associated molecular patterns [47]. Even though epithelium is a passive barrier protecting the underlying tissues in response to injury or infection, the epithelial cells can become a source of inflammatory mediators [48]. For instance, HSC2 cells respond to IL1β and TNFα with increased expression of IL6 [49]. However, whether butyrate and the other SCFA are capable of modulating the IL1β- and TNFα-induced expression of chemokines in oral epithelial cells remains unknown. 

Our first aim was to investigate how butyrate and other SCFAs can modulate the inflammatory response of RAW 264.7 and primary macrophages to LPS from *P. gingivalis* and fimbriae. The second aim was to evaluate the potential of SCFAs to lower the IL1β- and TNFα-induced inflammatory activity of gingival fibroblasts and oral HSC2 epithelial cells in vitro. 

## 2. Results

### 2.1. Butyrate Suppressed LPS-Induced IL6 and CXCL2 Expression in RAW 264.7 Cells

**Figure 1:**
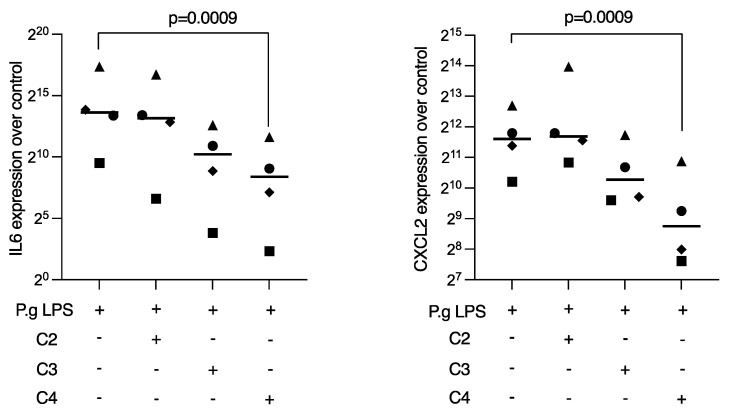
Butyrate suppressed *P. gingivalis* LPS-induced IL6 and CXCL2 expression in RAW 264.7 cells. RAW 264.7 cells were exposed for 24 h to 10 mM of acetate (C2), propionate (C3), and butyrate (C4) in the presence of 1 µg/mL *P. gingivalis* LPS. Each format shape represents data from individual experiments with the median. Statistical analysis was based on the Friedman test and corrected *p* values are indicated.

To determine whether SCFA can dampen the inflammatory response of macrophages to *P. gingivalis* LPS, RAW 264.7 cells were treated overnight with acetic, propionic, and butyric acid in the presence of the Toll-like receptor agonist. Reduced expression of IL6 and CXCL2 was noted when RAW 264.7 cells were exposed to butyric acid, while propionate and acetate failed to reach significance (Figure 1). Consistent results were observed at the protein level, where butyrate caused a reduction in *P. gingivalis* LPS-induced IL6 expression in RAW 264.7 cells (Table 1). Viability data are presents in Supplementary Table S1. 

### 2.2. Butyrate Reduced NF-κB Signaling in RAW 264.7 Cells

**Figure 2:**
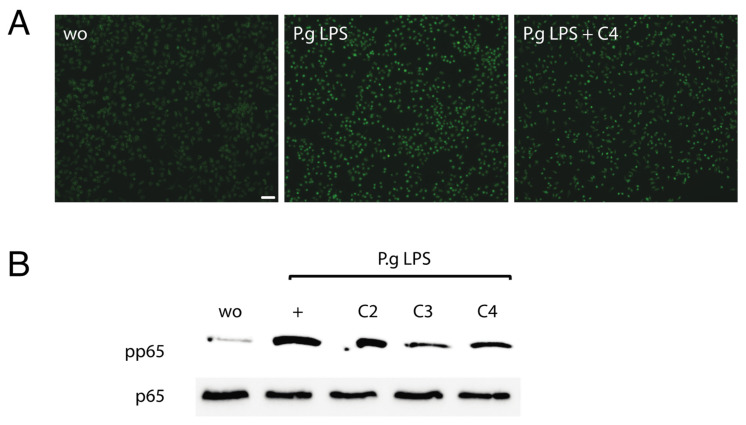
Butyrate reduced NF-κB signaling in RAW 264.7 cells. RAW 264.7 cells were exposed to 1 µg/mL *P. gingivalis* LPS (P.g LPS) with and without acetate (C2), propionate (C3), and butyrate (C4). (**A**) Immunofluorescence revealed that C4 reduced the intracellular translocation of NF-κB p65 into the nucleus. Green nuclei are positive-stained cells. (**B**) Western blot analysis showed that C3 and C4 lower the phosphorylation of p65. “wo” means “without” and represents unstimulated cells. Scale bar is 100 µm.

To confirm the anti-inflammatory activity of butyrate, NF-κB p65 immunofluorescence and Western blot analyses were performed. Consistent with its ability to lower IL6 and CXCL2 expression, butyrate reduced the *P. gingivalis* LPS-induced nuclear translocation of p65 in RAW 264.7 cells (Figure 2A). The presence of butyrate, but also propionate, suppressed the phosphorylation of p65 in RAW 264.7 cells when exposed to *P. gingivalis* LPS (Figure 2B). These results imply that butyrate and propionate reduce NF-κB signaling in RAW 264.7 cells.

### 2.3. Butyrate Suppressed Fimbriae and Saliva-Induced IL6 and CXCL2 Expression in Primary Macrophages

**Figure 3:**
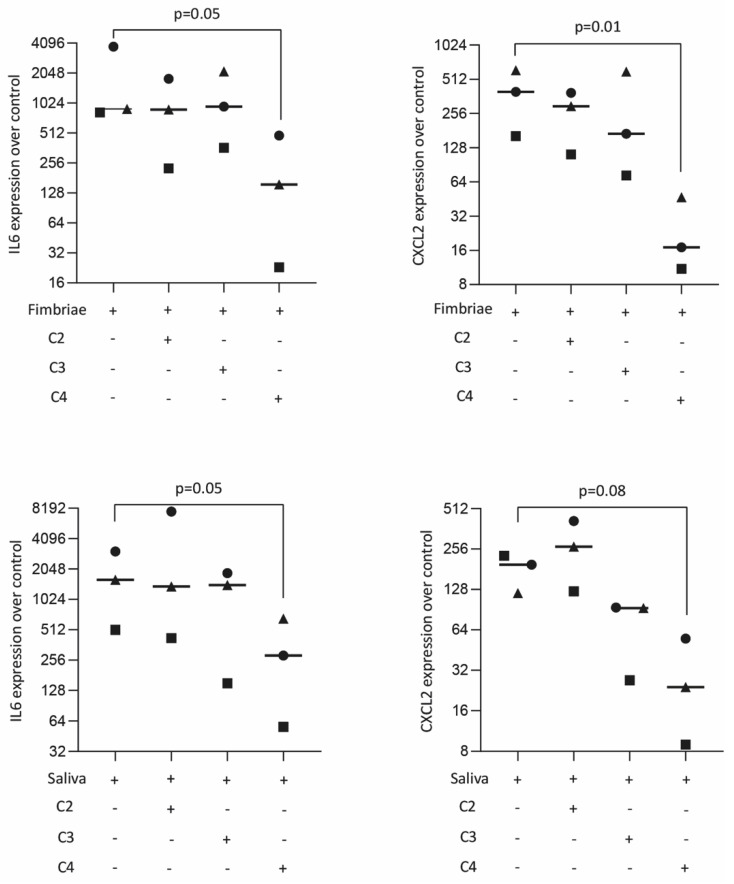
Butyrate suppressed *P. gingivalis* fimbriae and saliva-induced IL6 and CXCL2 expression in primary macrophages. Primary macrophages were exposed overnight to 10 mM of acetate (C2), propionate (C3), and butyrate (C4) in the presence of 100 µg/mL fimbriae and 5% saliva. Each format shape represents data from individual experiments with the median. Statistical analysis was based on the Friedman test and corrected *p* values are indicated.

We next confirmed the effects of butyrate observed with a cell line by using primary macrophages. Cells were stimulated with fimbriae from *P. gingivalis* LPS and saliva with and without acetate, propionate, and butyrate. Consistently, butyrate, but not propionate and acetate, significantly reduced the expression of IL6 and CXCL2 in primary macrophages in the presence of fimbriae from *P. gingivalis* LPS and saliva (Figure 3). Butyrate also lowered the expression of IL6 at the protein level (Table 2). These results provide strong evidence that butyrate was effective in reducing expression of IL6 and CXCL2 in primary macrophages.

### 2.4. Butyrate Reduced Inflammatory Mediators in Fibroblasts Exposed to IL1β and TNFα

**Figure 4:**
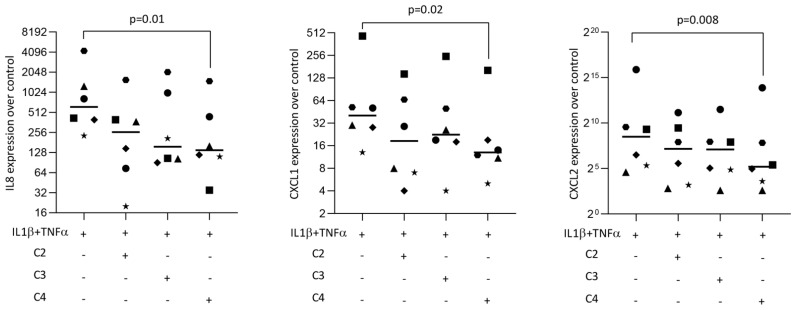
Butyrate, but not acetate or propionate, had significant anti-inflammatory effects in gingival fibroblasts. Cells were exposed to 10 mM of acetate (C2), propionate (C3), and butyrate (C4) in the presence of 10 ng/mL IL1β and TNFα. Each format shape represents data from individual experiments with the median. Statistical analysis was based on the Friedman test and corrected *p* values are indicated.

**Figure 5:**
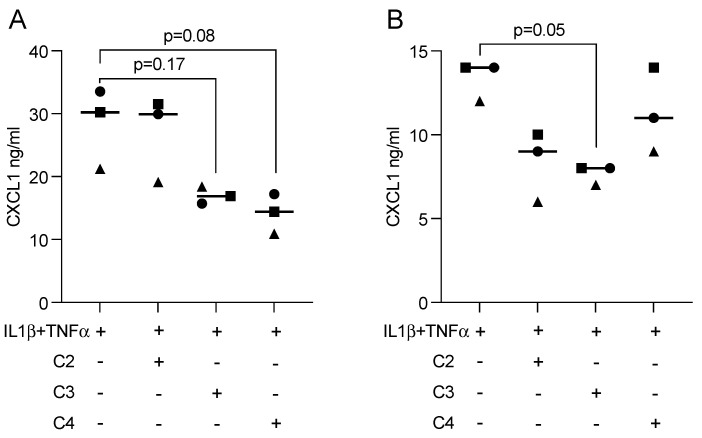
Immunoassay showed that propionate and butyrate suppress the expression of CXCL1 in (**A**) gingival fibroblasts and (**B**) HSC2 oral epithelial cells. Each format shape represents data from individual experiments with the median. Statistical analysis was based on the Friedman test and corrected *p*-values are indicated.

To examine whether SCFAs can modulate an inflammatory response in gingival fibroblasts, cells were treated overnight with acetic, propionic, and butyric acids in the presence of IL1β and TNFα. Statistical analysis revealed that butyrate was capable of significantly lowering the forced expression of IL8, CXCL1, and CXCL2 in gingival fibroblasts (Figure 4). Propionate and acetate also showed a trend towards anti-inflammatory activity but did not reach the level of significance. CXCL1 immunoassay using the supernatant from gingival fibroblasts confirmed the anti-inflammatory activity of butyrate (Figure 5A).

### 2.5. Propionate and Butyrate Reduced Inflammatory Mediators in HSC2 Cells Exposed to IL1β and TNFα

**Figure 6:**
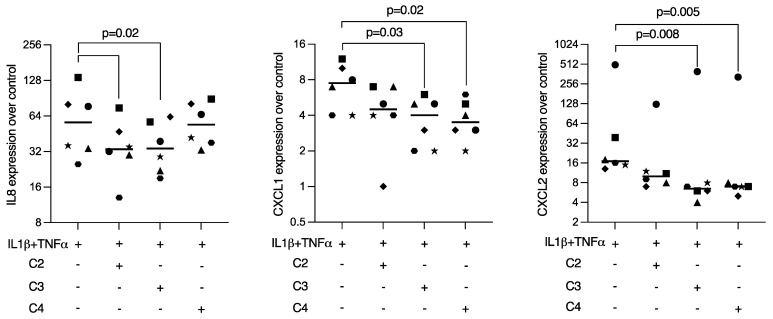
Propionate and butyrate suppressed the expression of CXCL1 and CXCL2 in HSC2 oral epithelial cells. Cells were exposed to 10 mM of acetate (C2), propionate (C3), and butyrate (C4) in the presence of 10 ng/mL IL1β and TNFα. Each format shape represents data from individual experiments with the median. Statistical analysis was based on the Friedman test and corrected *p* values are indicated.

To determine whether SCFAs can modulate anti-inflammatory response in oral epithelial cells, HSC2 cells were treated overnight with acetic, propionic, and butyric acids in the presence of IL1β and TNFα. Gene expression analysis revealed that butyrate reduced the expression of CXCL1 and CXCL2 in HSC2 cells exposed to IL1β and TNFα. However, only propionate significantly decreased the CXCL1 release into the supernatant of HSC2 cells (Figure 5B). With respect to IL8, only acetate and propionate were effective in HSC2 cells (Figure 6). Viability data are presents in Supplementary Table S1. 

### 2.6. Butyrate Decreased the Translocation of p65 in Gingival Fibroblasts and HSC2 Cells

**Figure 7:**
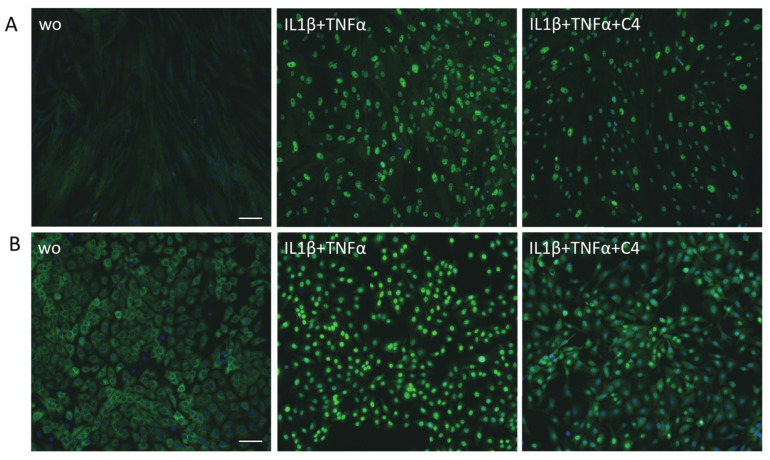
Butyrate decreased the nuclear translocation of p65 in gingival fibroblasts and HSC2 cells. (**A**) Gingival fibroblasts and (**B**) HSC2 oral epithelial cells were exposed to 10 ng/mL IL1β and TNFα with and without 10 mM butyrate (C4). Immunofluorescence of nuclear translocation of NF-κB p65 into the nucleus is indicated by green staining. Cells with no or low p65 nuclear translocation appear blue. “wo” means “without” and represents unstimulated cells. Scale bar is 100 µm.

To further confirm the ability of butyrate to attenuate NF-κB signaling, immunostaining of p65 was performed. Gingival fibroblasts and HSC2 oral epithelial cells were treated with butyrate, followed by exposure to IL1β and TNFα. Butyrate caused a visible reduction of p65 nuclear translocation in gingival fibroblasts (Figure 7A) and in HSC2 oral epithelial cells (Figure 7B). 

## 3. Discussion

SCFAs are well-recognized in periodontal research for two reasons: reason one is that propionic and butyric acid are metabolic by-products of anaerobic bacteria reaching approximately 10 and 3 mM, respectively, in the periodontal pocket of diseased subjects [15,16]. Reason two is that butyrate and propionate isolated from dental plaque extracts are harmful to various cell types related to the periodontium in vitro [8,9,10,11,12,13] but, on the other hand, have potential beneficial effects, particularly when acting systemically [17,18,19,20,21,22]. The current study provides further evidence to support a role for SCFAs in the control of the inflammatory response of periodontal cells. We initially established a bioassay showing that butyrate reduced the *P. gingivalis* LPS-induced expression of IL6 and CXCL2 in RAW 264.7 macrophage-like cells. We extend the original bioassay to primary macrophages where, again, butyrate significantly attenuated the *P. gingivalis* fimbriae- [28,29,30] and saliva-induced [31] expression of IL6 and CXCL2. Thus, butyrate, but not the other SCFAs, is a potent inhibitor of a forced inflammatory response of macrophages in vitro. With respect to gingival fibroblasts and HSC2 oral squamous epithelial cells exposed to IL1β and TNFα, the overall situation was less obvious. We can report that, in gingival fibroblasts, butyrate significantly reduced IL8, CXCL1, and CXCL2 expression, even though acetate and propionate showed a trend in this direction. Moreover, in oral epithelial HSC2 cells, butyrate and propionate significantly reduced CXCL1 and CXCL2 chemokines. Taking advantage of these bioassays, our data propose that butyrate in particular suppressed the in vitro inflammatory response in cell types relevant in periodontology. These findings are important because they add to the controversial discussion of whether or not SCFAs protect the periodontal tissue or push the catabolic process [8,39]. 

If we relate the findings to those of others working with macrophages, we have to acknowledge previous work showing that butyrate can attenuate inflammatory cytokine and nitric oxide production in RAW 264.7 cells exposed to LPS [24,25,26] and IFNγ [27]. Our data not only support findings observed with RAW 264.7 cells, we even strengthen the evidence by implementing *P. gingivalis* LPS-induced RAW 264.7 cells. We further extend the existing evidence by implementing fimbria isolated from *P. gingivalis* [28,29,30] and whole saliva [31] as an inducer of an inflammatory response in primary macrophages. Our data further underline the ability of butyrate, but not propionate or acetate, to significantly suppress the expression of inflammatory mediators, which were, in our case, IL6 and CXCL2. We can thus conclude that it is not necessarily the *E. coli* LPS-driven inflammatory response that is attenuated by butyrate; the more periodontitis-related agonists of inflammation, the fimbriae from *P. gingivalis* and saliva, also cannot exert their full pro-inflammatory potential in the presence of butyrate. 

With respect to the outcomes of others working with fibroblasts, the situation is less clear compared to what we know from macrophages. In gingival fibroblasts, butyrate and other SCFAs are considered drives of pathological tissue destruction and may promote progress of the periodontal disease state [15,23], including increasing proteolytic activity [35] and upregulating in vitro expression of TNFα in gingival fibroblasts [36] and even lung fibroblasts [50]. The concentrations of propionic and butyric acid accumulated in the gingival crevice were associated with disease severity and inflammation [15]. However, propionate reduced experimental arthritis and lowered the inflammatory response of synovial fibroblasts [37]. Butyrate further attenuated the inflammatory response in glomerular mesangial cells induced by high glucose and LPS [51] and, in myometrium cells and fetal membranes, stimulated with IL1β and TNFα [52]. Likewise, butyrate reduced cytokine and chemokine expression in co-culture methods of 3T3-L1 adipocytes and RAW 264.7 macrophages [53]. In line with the majority of in vitro studies, we observed a significant reduced expression of IL8, CXCL1, and CXCL2 in gingival fibroblasts exposed to IL1 and TNFα. Based on our findings, the anti-inflammatory activity of butyrate should not be ruled out. 

When compared to epithelial cells, butyrate was reported to destroy the epithelial barrier, pushing pyroptosis and provoking inflammatory responses by upregulation of IL8 and CCL2 [44]. Further, millimolar concentrations of butyrate were capable of inducing Ca9-22 cell death, increasing caspase-3 activity, phosphatidylserine redistribution, and bcl-2 down-regulation, suggesting that butyrate induced apoptosis [46]. On the other hand, in our study, butyrate was only effective in reducing CXCL1 and CXCL2 expression, while it was propionate that demonstrated attenuation of the IL1β- and TNFα-induced expression of IL8, CXCL1, and CXCL2 in HSC2 cells. Nevertheless, our data are consistent with findings that butyrate attenuates the excessive inflammation involved in the development of gastric mucosal ulcers induced by erosion due to acid in mice [54] and that butyrate inhibits LPS-induced cytokine production in mammary epithelial cells [55]. Butyrate further decreased TNFα-induced IL8 and CXCL2 expression in endothelial cells [56,57] and IL8 in the intestinal jejunal epithelial cell line J2 exposed to LPS [58]. Butyrate effectively inhibited CXCL10 expression in IFNγ- and TNFα-stimulated HT-29 intestinal epithelial cells [59]. Taken together, there is accumulating evidence for the in vitro anti-inflammatory activity of butyrate in bioassays with epithelial cells.

The clinical relevance of our findings is difficult to interpret. Nevertheless, our findings support the concept that oral health can be influenced by the oral microbiota, particularly when related to bacteria producing SCFAs. In support of this assumption, SCFAs were detected in gingival crevicular fluid subjects [15,16] partially originating from *F. nucleatum* [60]. According to our findings, butyrate and propionate are inhibitors of the inflammation-induced responses of macrophages, gingival fibroblasts, and also HSC2 oral epithelial cells, overall suggesting a beneficial effect of SCFAs for combat inflammatory tissue destruction. However, conclusions should not be drawn unless we understand the obvious discrepancy between the local and systemic action of the SCFAs, as the evidence of a pathological catabolic role of SCFAs accumulating in the periodontal pocket of severe periodontitis is undoubted; butyrate and propionate are detrimental for various cell types related to the periodontium in vitro [8,9,10,11,12,13]. Taken together, we have to consider the present research as a proof-of-principle study suggesting the potential anti-inflammatory activity of butyrate and propionate in the periodontium, under the premise that SCFAs in the mM range also exert periodontal tissue damage. 

The present study has limitations. For instance, in vitro models do not necessarily represent the clinical situation, and the selection of target genes, which were IL8, CXCL1, and CXCL2 in gingival fibroblasts and HSC2 cells, are indicator cytokines and do not represent the complex responses of cells in an inflammatory status. The same is true for macrophages exposed to LPS, which show robust expression of IL6 and CXCL2. It is also important to state that propionic and butyric at 10 and 3 mM, respectively, as observed in the subgingival microenvironment of diseased subjects [15,16], are harmful in vitro and may have affected the outcome of our in vitro study to some extent. Nevertheless, by selecting a 10 mM concentration of SCFAs, we tried to simulate the in vivo situation while keeping cell damage to a minimum by performing short-time experiments and the relevant controls to rule out that the anti-inflammatory effects were simply a consequence of cell death. 

Future research should consider the whole spectrum of changes at the transcription level, for instance, based on RNAseq. This approach would reveal the complexity of butyrate- and propionate-driven anti-inflammatory, and also destructive, activity. Our findings should be considered as one piece of a mosaic with the aim of better understanding how bacteria producing SCFAs might play an active role in controlling tissue destruction in periodontitis and also periimplantitis. 

## 4. Materials and Methods

### 4.1. Primary Macrophages and RAW 264.7 Macrophage-like Cells

For the isolation and culture of murine bone marrow-derived macrophages, 6–8 week old BALB/c mice (Animal Research Laboratories, Himberg, Austria) were purchased. Bone marrow cells were collected from the femora and tibiae and grown for 5 days in Dulbecco’s Modified Eagle Medium (DMEM; Sigma Aldrich, St. Louis, MO, USA), supplemented with 10% fetal calf serum (FCS; Capricorn Scientific GmbH, Ebsdorfergrund, Germany) and antibiotics (Sigma Aldrich, St. Louis, MO, USA) supplemented with 20 µg/mL macrophage colony-stimulating factor (M-CSF; ProSpec-Tany TechnoGene Ltd., Rehovot, Israel). RAW 264.7 macrophage-like cells (LGC Standards, Wesel, Germany) were expanded in growth medium and seeded at 1 × 10^6^ cells/cm^2^ into 24-well plates (CytoOne, Starlab International, Hamburg, Germany). The cells were cultured in the growth medium at 37 °C, 5% CO_2_, and 95% humidity. Serum-free conditions were used during cell stimulation. 

### 4.2. Gingival Fibroblasts and HSC2 Oral Epithelial Cells 

Gingival fibroblasts were prepared using explant cultures of human gingiva harvested from extracted third molars of patients who had given informed and written consent. The Ethics Committee of the Medical University of Vienna (EK NR 631/2007) Vienna, Austria, approved this protocol. Human oral epithelial HSC2 cells ere kindly provided by Prof. Rausch-Fan from the Medical University of Vienna, Vienna, Austria. Gingival fibroblasts and HSC2 cells were cultured in the growth medium and seeded at a concentration of at least 30,000 cells/cm^2^ onto culture dishes one day prior to stimulation. Serum-free conditions were used during cell stimulation.

### 4.3. Viability Assay

Cells were incubated with different concentrations of acetate, propionate, and butyrate (Sigma-Aldrich, St. Louis, MO, USA) or serum-free medium in 96-well plates. After 24 h, a final concentration of 0.5 mg/mL of an MTT (3-(4,5-dimethythiazol-2-yl)-2,5-diphenyltetrazolium bromide; Sigma-Aldrich, St. Louis, MO, USA) solution was added to each well of the microtiter plate for 2 h at 37 °C. After medium removal, formazan crystals were solubilized with dimethyl sulfoxide. Assessment of optical density was carried out for 570 nm and expressed as the percentage of unstimulated controls. Data are presents as Supplementary Table S1. 

### 4.4. Cell Stimulation

RAW 264.7 and primary macrophages were stimulated with 10 mM of SCFAs overnight in serum-free medium with the addition of 1 µg/mL LPS from *Porphyromonas gingivalis* (Sigma-Aldrich, St. Louis, MO, USA), 5% sterile-filtered saliva [31], and 100 µg/mL fimbriae from *P. gingivalis* (CusaBio Technology LLC, Houston, TX, USA). In the case of gingival fibroblasts and HSC2 cells, inflammation was simulated with 10 ng/mL IL1β and TNFα (ProSpec-Tany TechnoGene Ltd., Rehovot, Israel).

### 4.5. qRT-PCR Analysis and Immunoassay

Total RNA was isolated with the ExtractMe total RNA kit (Blirt S.A., Gdańsk, Poland). Reverse transcription (RT) was performed with the LabQ FirstStrand cDNA Synthesis Kit (LabQ, Labconsulting, Vienna, Austria). The reverse transcription-polymerase chain reaction was done (LabQ, Labconsulting, Vienna, Austria) on a CFX Connect™ Real-Time PCR Detection System (Bio-Rad Laboratories, Hercules, CA, USA). Primer sequences are shown in Table 3 and Table 4. The mRNA levels were calculated by normalizing to the housekeeping genes GAPDH using the ΔΔCt method. Expressing levels in unstimulated cells are normalized and, thus, calculated as 1. The immunoassay was performed with the mouse IL6 and human CXCL1 Quantikine ELISA kit (R&D Systems, Minneapolis, MN, USA).

### 4.6. Immunofluorescence

RAW 264.7 cells were plated onto Millicell^®^ EZ slides (Merck KGaA, Darmstadt, Germany) and treated with 10 mM of SCFAs overnight before being exposed to *P. gingivalis* LPS for 40 min. Gingival fibroblasts and HSC2 cells were treated with 10 mM of SCFAs for one hour before being exposed to IL1β and TNFα for 40 min. Cells were then fixed in 4% paraformaldehyde and blocked in 5% bovine serum albumin and 0.3% Triton X-100 in phosphate-buffered saline at room temperature. After permeabilization with 0.1% Triton X-100, cells were incubated with nuclear factor kappa B (NF-κB) p65 antibody (#8242, Cell Signaling Technology, Danvers, MA, USA) overnight at 4 °C and visualized with a Alexa Fluor^®^ 488-conjugated secondary antibody (#4412S, Cell signaling Technology, Danvers, MA, USA). Images were captured under a fluorescent microscope (Echo Revolve MTEL2LL/A, San Diego, CA, USA). 

### 4.7. Western Blot

After stimulation with SCFAs for 24 h and *P. gingivalis* LPS, the lysates from RAW 264.7 cells were separated with SDS-PAGE and transferred onto PVDF membranes (Roche Diagnostics, Mannheim, Germany). Membranes were blocked with 5% skim milk. The binding of the first antibodies—phospho-NF-κB p65 antibodies (#S536, Cell Signaling Technology) and NF-κB p65 antibody (#D14E12, Cell Signaling Technology)—was detected with the second antibody labelled with peroxidase (#CS-7074, Cell Signaling Technology). After their exposure to the Clarity Western ECL Substrate (Bio-Rad Laboratories, Inc., Hercules, CA, USA), chemiluminescence signals were visualized with the ChemiDoc imaging system (Bio-Rad Laboratories).

### 4.8. Statistical Analysis

The experiments were repeated at least three times. Dot plots show the data from all independent experiments with the median. Statistical analysis was based on the Friedman test with correction for multiple comparisons. Analyses were performed using Prism v8 (GraphPad Software, La Jolla, CA, USA). Significance was set at *p* < 0.05.

## 5. Conclusions

In conclusion, we show here that butyrate and propionate, in particular, show potent and robust anti-inflammatory activity based on in vitro models of macrophages, gingival fibroblasts, and oral epithelial cells. These findings are a solid basis to further refine our knowledge on how SCFAs produced by bacteria, but maybe also applied pharmacologically, might help to prevent inflammatory tissue destruction and even support the regeneration process.

## Figures and Tables

**Table 1:** Butyrate suppressed *P. gingivalis* LPS-induced IL6 production of RAW 264.7 cells. RAW 264.7 cells were exposed for 24 h to 10 mM of butyrate (C4) in the presence of 1 µg/mL *P. gingivalis* LPS. Data represent the concentration of IL6 in pg/mL in the supernatant from two independent experiments.

	wo	P.g LPS	P.g LPS + C4
Experiment 1	167	880	221
Experiment 2	113	789	167

**Table 2:** Butyrate suppressed *P. gingivalis* fimbriae and saliva-induced IL6 levels in primary macrophages. Primary macrophages were exposed overnight to 10 mM of butyrate (C4) in the presence of 100 µg/mL fimbriae from *P. gingivalis* and 5% saliva. Data represent the concentration of IL6 in pg/mL in the supernatant from two independent experiments.

	wo	Fimbriae	Fimbriae + C4	Saliva	Saliva + C4
Experiment 1	481	4872	706	15,469	1234
Experiment 2	320	1691	565	1827	442

**Table 3:** Mouse primers sequences.

Primer	Sequence Forward	Sequence Reverse
IL6	gctaccaaactggatataatcagga	ccaggtagctatggtactccagaa
CXCL2	catccagagcttgagtgtgacg	ggcttcagggtcaaggcaaac
GAPDH	aactttggcattgtggaagg	ggatgcagggatgatgttct

**Table 4:** Human primers sequences.

Primer	Sequence Forward	Sequence Reverse
IL8	aacttctccacaaccctctg	ttggcagccttcctgatttc
CXCL1	tcctgcatcccccatagtta	cttcaggaacagccaccagt
CXCL2	cccatggttaagaaaatcatcg	cttcaggaacagccaccaat
GAPDH	aagccacatcgctcagacac	gcccaatacgaccaaatcc

## Data Availability

All data are made available on demand.

## Supplementary Materials

The following supporting information can be downloaded at: https://www.mdpi.com/article/10.3390/ijms231911006/s1.
