# Supplementary material for: Proof-of-Principle Study Suggesting Potential Anti-Inflammatory Activity of Butyrate and Propionate in Periodontal Cells
Source: Int J Mol Sci. 2022 Sep 20;23(19):11006. doi: 10.3390/ijms231911006 (PMC9570314; doi:10.3390/ijms231911006)
Supplement: Supplementary file 1 [file ijms-23-11006-s001.zip › ijms-1910099-supplementary.pdf]

Supplemental Files

To expand the evaluation of the impact of SCFAs cell viability was performed, an MTT assay. For this, SCFAs were used in three different concentrations: 1mM, 10mM and 20mM. The data obtained were inserted in Supplemental Table 1, in which it is observed that RAW 264.7 were more sensitive to butyric acid, with 10mM being the maximum non-toxic concentration. On the other hand, even the concentration of 20mM SCFA is not toxic for gingival fibroblasts and HSC2 oral epithelial cells. Thus, as it was not toxic in any of the cells analyzed, it was decided to use the concentration of 10mM of SCFAs for consecutive analyses.

Cell type	RAW 264.7			Primary macrophages		
Concentration	*Acetate*	*Propionate*	*Butyrate*	*Acetate*	*Propionate*	*Butyrate*
20mM	99.0±20.9	69.8±24.1	51.5±36.7	101.1±6.1	90.8±4.8	90.1±2.3
10mM	94.3±18.6	90.5±18.7	56.8±34.3	111.3±16.3	99.5±6.5	93.0±3.5
1mM	91.0±43.3	108.0±24.4	52.7±34.8			97.3±7.8

Cell type	Gingival fibroblasts			HSC2 oral epithelial cells		
Concentration	*Acetate*	*Propionate*	*Butyrate*	*Acetate*	*Propionate*	*Butyrate*
20mM	104.0±4.2	111.5±9.1	103.4±2.9	119.5±34.6	108.5±29.9	62.0±27.7
10mM	97.5±0.7	96.5±9.1	105.1±11.3	111.0±32.5	114.0±33.9	95.0±5.9
1mM	99.0±15.5	97.0±1.4	106.4±10.3	92.5±12.0	88.0±9.8	107.0±12.6

Supplemental Table S1. *Cell viability of RAW 264.7, primary macrophages, gingival fibroblasts and HSC2 oral epithelial cells exposed to different concentrations of SCFA.* Cell viability is represented by formazan production indicated in percentage of unstimulated controls ± SD. Cells maintained their viability with up to 10 mM of SCFA.
